# Targeting protein quality control pathways in breast cancer

**DOI:** 10.1186/s12915-017-0449-4

**Published:** 2017-11-16

**Authors:** Sara Sannino, Jeffrey L. Brodsky

**Affiliations:** Department of Biological Sciences, University of Pittsburgh, A320 Langley Hall, 4249 Fifth Ave, Pittsburgh, PA 15260 USA

## Abstract

The efficient production, folding, and secretion of proteins is critical for cancer cell survival. However, cancer cells thrive under stress conditions that damage proteins, so many cancer cells overexpress molecular chaperones that facilitate protein folding and target misfolded proteins for degradation via the ubiquitin-proteasome or autophagy pathway. Stress response pathway induction is also important for cancer cell survival. Indeed, validated targets for anti-cancer treatments include molecular chaperones, components of the unfolded protein response, the ubiquitin-proteasome system, and autophagy. We will focus on links between breast cancer and these processes, as well as the development of drug resistance, relapse, and treatment.

## Breast cancer subtypes and cellular protein quality control pathways

Breast cancer is a complex and heterogeneous disease that remains the most prevalent cancer diagnosed in women and is responsible for the greatest proportion of cancer-related deaths in women [1]. Breast cancers are divided into different subtypes depending on the expression of hormone receptors, including estrogen receptor (ER), progesterone receptor (PR), and epidermal growth factor receptor 2 (HER2 or erbB2) [2, 3]. Luminal breast cancers are characterized by ER overexpression and fall into the luminal A or B class, in which, respectively, either both ER and PR are overexpressed or ER is overexpressed and HER2 may also be overexpressed. HER2-positive breast cancer, in which HER2 is overexpressed, represents another subtype, and can be diagnosed at a younger age compared to the luminal A and luminal B cancers. Finally, triple negative breast cancers (TNBC) are ER-, PR-, and HER2-negative [2, 3].

ER-positive breast cancer groups are especially prevalent and mainly afflict postmenopausal women because luminal cells become more sensitive to estrogen (17β-estradiol or E_2_) levels as a result of hormonal fluctuations [4]. Activation of the ER signaling cascade stimulates cell division, tumor growth, and metastasis. Therefore, ER-positive patients are initially treated with anti-estrogen therapies [5] (Table 1). Tamoxifen was one of the first FDA approved drugs used to treat these patients, is a non-steroid inhibitor of the receptor, and blocks downstream signaling [6, 7]. However, in many tamoxifen treated patients, ER activation was still detected, highlighting the demand for improved compounds and new targets [8]. In fact, since tamoxifen was approved, different ER-targeted drugs were introduced that downregulate the receptor, induce receptor degradation, or attenuate ER signaling (Table 1) [9–12]. Nevertheless, endocrine-treatment resistance remains one of the leading causes of breast cancer mortality [12, 13].

**Table 1:** Examples of drugs used in ER-positive breast cancer treatment

Drug name	Notes	Developmental stage	References
Fulvestrant (Faslodex)	Induces ER degradation, nonsteroidal selective estrogen receptor degrader (SERD)	FDA approved	[288–292]
Tamoxifen	Nonsteroidal selective estrogen receptor modulator (SERM)	FDA approved	[6, 288, 293–295]
Raloxifene hydrochloride	Nonsteroidal selective estrogen receptor modulator (SERM)	FDA approved	[296–301]
Toremifene (Fareston)	Nonsteroidal selective estrogen receptor modulator (SERM)	FDA approved	[301, 302]
ARN-810	Nonsteroidal selective estrogen receptor degrader (SERD)	Clinical trial	[303, 304]
AZD9496	Nonsteroidal selective estrogen receptor degrader (SERD)	Clinical trial	[305, 306]
RAD1901	Nonsteroidal selective estrogen receptor degrader (SERD)	Clinical trial	[307–309]
Letrozole (Femara)	Nonsteroidal inhibitor of estrogen synthesis (aromatase inhibitor)	FDA approved	[310–313]
Anastrozole (Arimidex)	Nonsteroidal inhibitor of estrogen synthesis (aromatase inhibitor)	FDA approved	[314–317]
Exemestane (Aromasin)	Nonsteroidal inhibitor of estrogen synthesis (aromatase inhibitor)	FDA approved	[318–321]
BEZ235	Dual inhibitor of PI3K and mTOR	Clinical trial	[322–325]
SAR245409	PI3K inhibitor	Clinical trial	[285, 313]
Taselisib	Class I PI3K alpha inhibitor	Clinical trial	[326–328]
Buparlisib	PI3K inhibitor, competes for the ATP binding	Clinical trial	[63, 326]
Venetoclax (*ABT-199)*	Bcl-2 inhibitor	Clinical trial	[329, 330]
Everolimus (Afinitor)	Inhibitor of mTORC1 used both in luminal A and in HER2-positive tumors	FDA approved	[277–279, 283]
Temsiroliums	Inhibitors of mTORC1 used in luminal A, TNBC, and HER2-positive tumors	Clinical trial	[331]
Vorinostat (SAHA)	Global inhibitor of HDAC	Clinical trial	[332–334]
Entinostat	Inhibitor of HDAC1 and HDAC3	Clinical trial	[335, 336]
Panobinostat	Specific inhibitor of HDAC	Clinical trial	[337–339]
Rapamycin	mTOR inhibitor	HER- and ER-positive breast cancer cells	[340–344]
SNIPER(ER)	PROTAC-mediated ER degradation in breast cancer cells	ER-positive breast cancer cells	[98, 345]
BHPI	Modulator of ER-dependent UPR response	ER-positive breast cancer cells	[168, 187, 188, 346]
MAb159	Monoclonal antibody against BiP	ER-positive breast cancer cells	[222]
Plumbagin	BiP downregulator, induces BIK levels	ER-positive breast cancer cells	[220]
Epigallocatechin gallate (EGCG)	Inhibits cellular oxidation and DNA methyltransferase to block EGFR and HER2 activation and can induce UPR response by binding BiP	TNBC, ER- and HER2-positive breast cancer cells	[347–352]
Resveratrol	Activates SIRT-1 and inhibits TNF-induced activation of NFkB. Used in combination with bortezomib, reduces cell viability through autophagy inhibition	ER- and HER2-positive breast cancer cells	[353, 354]
Hydroxychloroquine	Autophagy inhibitor, suppresses lysosomal acidification	Clinical trial	[167, 225]

The HER2 amplified class represents 15–20% of all the breast cancers and patients with this variant have benefited from significant clinical successes [3, 14]. HER2 plays a key role in cellular hemostasis and tissue development (such as during epithelial mammary gland organization [15]). There are four members of the HER tyrosine kinase family, HER, HER2, HER3, and HER4 (also known as EGFR, erbB2, erbB3, and erbB4, respectively), and maintenance of proper receptor numbers at the plasma membrane is critical for signal transduction [15, 16]. Upon ligand binding, the receptors homo- or hetero-dimerize, inducing an intracellular signaling cascade [17]. Receptor endocytosis also represents a key regulatory event [16]. While many ligands for EGRF, HER3, and HER4 are known, no HER2 ligand has been identified. Moreover, in contrast to the other family members, HER2 is the preferred dimerization partner of the other HERs, in particular HER3, which activates downstream PI3K/Akt signaling [18–21]. Preclinical studies supported a key function for HER3 in promoting the growth of HER2-positive breast cancer cells [22], and these patients are now prime candidates for trastuzumab (Herceptin, a monoclonal antibody against HER2), perstuzumab (Perjeta, a HER2 and HER3 dimerization inhibitor), and lapatinib (Tykerb, a tyrosine kinase inhibitor), as well as for other compounds directed against HER2 and/or downstream kinases (Table 2). Yet despite the apparent specificity of these drugs, some patients remain resistant to these treatments [23], mainly due to metastasis and receptor mutations, which reduces patient survival and increases tumor relapse [24]. Moreover, ~ 10% of metastatic luminal breast cancers metastasize to the brain, and in this case the only treatment options include chemotherapy, radiation, and/or surgery [12]. Therefore, the identification of markers during early stage therapy is also of fundamental importance.

**Table 2:** Examples of drugs used in HER2 breast cancer treatment

Drug name	Notes	Developmental stage	References
Trastuzumab (Herceptin)	Monoclonal antibody against HER2	FDA approved	[355–360]
Ado trastuzumab emtansine (T-DM1)	Bifunctional antibody-drug (trastuzumab linked to ematansine, DM1). Binds HER2 and inhibits microtubule assembly/disassembly	FDA approved	[361–364]
Eetumaxomab	Monoclonal antibody with CD3 and HER2 recognition sites	Clinical trial	[365, 366]
Pertuzumab (Perjeta)	Recombinant humanized antibody against domain II of HER2	FDA approved	[284, 367, 368]
MM-111	Antibody against HER2-HER3 dimers	Clinical trial	[369, 370]
Lapatinib (Tykerb)	Irreversible tyrosine kinase inhibitor in luminal B cancers	FDA approved	[371, 372]
Afatinib	Irreversible pan-HER tyrosine kinase inhibitor	Clinical trial	[373–375]
Canertinib	Irreversible tyrosine kinase inhibitor	Clinical trial	[19, 376, 377]
Neratinib	Irreversible pan-HER tyrosine kinase inhibitor, effective against EGFR, HER2, and HER4	Clinical trial	[378–381]
Gefitinib	EGFR tyrosine kinase inhibitor	Clinical trial	[382, 383]
Erlotinib hydrochloride (Erlotinib)	Reversibly binds to the intracellular catalytic domain of EGFR; used also in TBNC cancers	Clinical trial	[383–385]
Sapitinib	Tyrosine kinase inhibitor effective in luminal B resistant cells	Clinical trial	[380, 386]
Sorafenib	Blocks the enzyme RAF kinase, inhibiting cancer cell proliferation and autophagy induction	Clinical trial	[63, 387–389]
Sildenafil citrate	Selectively inhibits cyclic guanosine monophosphate (cGMP)-specific type 5 phosphodiesterase	Clinical trial	[63]
MM-121	Human monoclonal antibody against HER3	Clinical trial	[267, 390–392]
MM-302	Doxorubicin encapsulated within liposomes, and conjugated to a monoclonal antibody against HER2. Inhibits HER2 and topoisomerase II	Clinical trial	[393, 394]
ARRAY-380	Reversible selective HER2 inhibitor	Clinical trial	[395]
TAK-285	HER2-EGFR tyrosine kinase inhibitor	HER2-positive breast cancer cells	[396–399]
Everolimus (Afinitor)	Inhibitor of mTORC1 used both in luminal A and in HER2-positive tumors	FDA approved	[277–279, 283]
Temsiroliums	Inhibitor of mTORC1 used in luminal A, TNBC, and HER2-positive tumors	Clinical trial	[331]
GDC-0941	PI3K inhibitor	Clinical trial	[400, 401]
SAR245408	PI3K inhibitor	Clinical trial	[402, 403]
17-AAG	Hsp90 inhibitor	Clinical trial	[404–406]
Retaspinmycin (IPI-504)	Hsp90 inhibitor	Clinical trial	[406–408]
Genetespib	Hsp90 inhibitor used in metastatic HER2 breast cancers	Clinical trial	[409–412]
Pazopanib	inhibitor of VEGFRs able to inhibit Hsp90 ATPase activity	Clinical trial	[63, 413, 414]
SNX-2112	Hsp90 inhibitor, effective in HER2 and luminal B breast cancers	Clinical trial	[83, 280, 415]
Geldanamycin	Hsp90 inhibitor	HER2-positive breast cancer cells	[20, 109, 114–116]
KIN001-51	HER3 binder, impairs dimerization	HER2-positive breast cancer cells	[416, 417]
TX1-85-1	Induces HER3 degradation by covalent binding to a residue in the receptor	HER2-positive breast cancer cells	[100, 383]
TX2-121-1	Derivate of TX1-85-1 linked to adamantane group. Induces HER3 degradation	HER2-positive breast cancer cells	[100, 383]
Patritumab (AMG 888)	Monoclonal antibody directed against the ligand-binding pocket of HER3	Clinical trial	[418]
MEHD7945A	Monoclonal antibody directed against EGFR and HER3	Clinical trial	[419, 420]
Pilaralisib	Pan-class I PI3K inhibitor	HER2-positive breast cancer cells	[224, 421]
liposomal paclitaxel	Inhibits tubulin assembly/disassembly	FDA approved	[224, 421, 422]
hydroxychloroquine	Autophagy inhibitor, suppresses lysosomal acidification	Clinical trial	[167, 225]
Eeyarestatins	p97 inhibitor	HER2-positive breast cancer cells	[81, 85]
NMS-873	p97 inhibitor	HER2-positive breast cancer cells	[81]
HA15	Inhibitor of BiP ATPase activity	HER2-positive breast cancer cells	[173]

Over the past several years, alternative approaches to treat breast cancer have been pursued, focusing on the regulation of protein homeostasis (“proteostasis”) and stress response pathways [25–30]. These approaches include modulation of protein degradation pathways mediated by the proteasome and autophagy, and the regulation of cellular stress responses, with particular attention paid to the unfolded protein response (UPR).

The ubiquitin-proteasome pathway utilizes a cascade of E1 ubiquitin-activating enzymes, E2 ubiquitin-conjugating enzymes, and E3 ubiquitin ligases [31–34]. Once a protein substrate has acquired at least four ubiquitin species, the substrate is delivered to the 26S proteasome, which houses three unique protease activities to destroy protein substrates. Protein ubiquitylation can be reverted by a family of proteins called deubiquitylating enzymes (DUBs) that are involved in removing mono- and poly-ubiquitin chains from proteins, thereby changing the fates of their targets and maintaining the pool of free ubiquitin [35–37]. In contrast, autophagy is a process in which damaged proteins are encapsulated and degraded in double-membrane structures, called autophagolysosomes [38–42]. During canonical autophagy (formally called macroautophagy), an isolation membrane encloses a portion of the cytoplasm containing misfolded proteins, protein aggregates, and even organelles to form a vesicle called the autophagosome. The contents of the autophagosomes are degraded upon subsequent fusion with lysosomes [2, 38–41, 43, 44].

The proteasome pathway has been effectively targeted in select cancers, primarily because protein folding is an inherently slow, energy expensive, and inefficient pathway. Therefore, each cellular compartment is equipped with a variety of molecular chaperones and folding enzymes that coordinate protein folding; however, if protein folding is delayed or compromised, aberrant proteins are instead targeted to the ubiquitin-proteasome system [45–48]. Proteasome inhibitors such as carfilzomib (Kyprolis) and bortezomib (Velcade) are especially effective for the treatment of multiple myeloma [49, 50] because myeloma cells produce high levels of misfolded or unassembled immunoglobulin subunits [51–55]. Also, due to their higher proliferation rate, cancer cells require increased levels of ATP for enhanced protein production and there is a heightened demand on cellular protein folding pathways. Cancer cells can also become hypoxic, hypoglycemic, and acidic, which perturbs cellular hemostasis and—in particular—secretory protein folding in the endoplasmic reticulum [28, 56, 57]. Moreover, most cancer cells contain DNA duplications, deletions, inversions, and translocations, as well as altered chromosome numbers (aneuploidy) [58], which alters the stoichiometries of protein complexes. Consequently, many proteins become “orphaned” and misfold [59, 60]. To overcome the ensuing onslaught of these misfolded proteins, cancer cells also overexpress multiple chaperones to maintain cellular homeostasis [33, 48, 61–63].

**Fig. 1.**
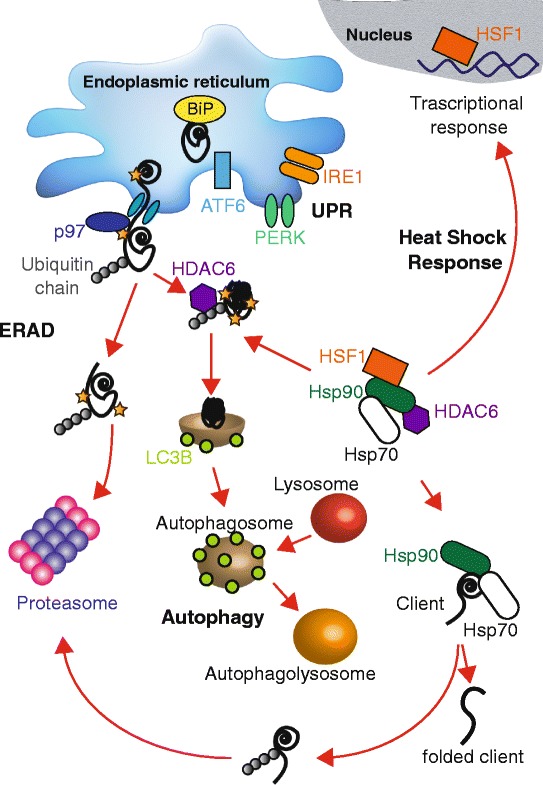
Schematic representation of secretory protein folding and quality control, the unfolded protein response pathway, and the heat shock response. In the endoplasmic reticulum, unfolded proteins (*black lines*) can be recognized and bound by chaperones, such as BiP (*yellow circles*). The increase in the concentration of BiP-unfolded protein complexes in the endoplasmic reticulum favors induction of the unfolded protein response (*UPR*). The UPR is regulated by ATF6 (*light blue rectangle*), PERK (*green dimers*), and IRE1 (*orange dimers*), which reside in the endoplasmic reticulum membrane. Upon activation, the UPR can increase cellular folding capacity by increasing chaperone synthesis, inducing endoplasmic reticulum expansion, and increasing the concentration of components of the endoplasmic reticulum associated degradation (*ERAD*) machinery. During ERAD, unfolded proteins in the endoplasmic reticulum are recognized, ubiquitylated by E3 ubiquitin ligases, and retrotranslocated via the action of p97 (*blue circle*), an AAA-ATPase, to the cytosol where they are degraded by the proteasome. Misfolded, aggregation-prone proteins, protein aggresomes, and damaged organelles can alternatively be targeted for autophagy via encapsulation in double membrane vesicles known as autophagosomes (*light brown vesicles*). LC3BII is an established marker of cellular autophagy and is associated with autophagosome membranes (*light green circles*), and proteins can be directed to autophagy degradation via HDAC6 (*purple hexagon*). Upon fusion with lysosomes (*red vesicles*), the material incorporated in the autophagolysosome is degraded (*orange vesicles*). In the absence of stress, HSF1, HSP90, HSP70, and HDAC6 can form a complex in the cytoplasm. During stress (for example, an increase in the concentration of unfolded protein or heat), HSF1 (*orange rectangle*) can translocate to the nucleus and induce the transcription of other proteins, like chaperones, to increase the cellular folding capacity. This is known as the heat shock response. At the same time, Hsp90 and Hsp70 (*green* and *white rounded rectangles*, respectively) are involved in cytoplasmic protein folding, dictating the fate of their clients. If the client fails to attain its final conformation, it will be ubiquitylated and degraded by the proteasome

In the remainder of this review, we will discuss the links between protein folding, degradation, and transport and breast cancer survival with an emphasis on the different mechanisms involved in controlling secretory protein folding. The highlighted mechanisms include the endoplasmic reticulum-associated degradation (ERAD) pathway, the unfolded protein response (UPR), and the autophagy pathway (Fig. 1). Finally, we will emphasize critical areas for further research that may improve breast cancer treatments.

## Proteasome-mediated degradation as a possible therapeutic target

Approximately one-third of all proteins in eukaryotes are targeted to the endoplasmic reticulum [45, 46, 64]. Nevertheless, proteins entering this compartment—which represents the first step in the secretory pathway—may misfold due to stochastic errors in the folding pathway or as a result of cellular stress. Cellular attempts to temper protein toxicity in the secretory pathway are based on two different mechanisms: first, the UPR can be induced, which increases the chaperone reservoir in the endoplasmic reticulum and induces endoplasmic reticulum expansion, and second, misfolded proteins can be targeted for degradation to either the lysosome (via autophagy) or to the proteasome [31, 65, 66]. The process that targets unfolded proteins in the endoplasmic reticulum to the proteasome is known as endoplasmic reticulum-associated degradation, or ERAD.

ERAD is a multistep pathway, and many of the components required for this event are induced by the UPR [66, 67]. ERAD substrates are selected by a pool of chaperones and lectins and, in the case of soluble luminal substrates, are partially transported across the membrane to expose their cytoplasmic domains, which allows for ubiquitylation [34, 36, 68, 69]. The acquisition of a poly-ubiquitin chain recruits the AAA^+^ ATPase p97, which “retrotranslocates” ERAD substrates from the endoplasmic reticulum [70–73]. Once retrotranslocated, ubiquitylated substrates are degraded by the 26S proteasome. p97 is not only involved in ERAD substrate retrotranslocation but also modulates protein trafficking in the secretory pathway, mitochondrial-associated protein degradation, the degradation of aberrant ribosome-associated proteins, chromatin remodeling, and autophagy, each of which is also associated with cancer [74–80]. Recently, HER2-positive breast cancer cells were shown to have elevated levels of p97, suggesting in one study that ERAD contributes to cancer cell survival [81]. Based on these data and the importance of p97 during the disposal of misfolded proteins, p97 inhibitors are being examined for possible therapeutic applications in cancer cells. The eeyrestatins (Eer I and II) were the first generation of p97 inhibitors and in several studies were shown to reduce ERAD efficiency [82–85]. HER2-positive cells are more sensitive to both eeyarestatin and NMS-873, a refined p97 inhibitor, compared to other breast cancer cells that did not overexpress HER2 [81]. Recently a new p97 inhibitor was characterized (CB-5083) as a potential anti-cancer drug both in multiple myeloma and in various solid tumors, including lung and colorectal carcinoma [86]. Thus far, clinical trials using this compound in breast cancer patients have not yet begun.

Human epidermal growth factor receptors, or HER family members, are selected for endosomal sorting and lysosomal degradation from the plasma membrane, and as noted above the regulation of receptor numbers at the plasma membrane is crucial for cell survival [87, 88]. However, the levels of HER3 that ultimately reside at the plasma membrane are also regulated in the endoplasmic reticulum by ERAD in a p97-dependent manner. In fact, HER3 is ubiquitylated by an E3 ubiquitin ligase known as Nrdp1 in the endoplasmic reticulum [88, 89]. Nrdp1 also ubiquitylates HER3 at the plasma membrane, which is required for endocytosis and lysosomal sorting/degradation [88, 90, 91]. Consistent with the importance of properly regulating the levels of HER3-containing heterodimers, Nrdp1 is suppressed in 57% of breast cancer tissues [92–94]. This E3 ubiquitin ligase also interacts with HER4 in breast cancer cells [95]. One study suggested that, after endoplasmic reticulum stress and UPR induction, Nrdp1 becomes trapped in tubular structures, which impairs HER3 degradation [96]. These data highlight how ERAD, plasma membrane protein degradation, and stress may be linked to signal transduction pathways and proteostasis in cancer cells.

Based on its central role in protein quality control, compromising other early steps in the proteasome-dependent degradation pathway might deplete cancer cells of their oncogenic signaling potential and provide another therapeutic route. To this end, PROTACs were developed, which are heterobifunctional molecules that contain a binding motif for the target of interest and for an E3 ubiquitin ligase. Addition of the molecule induces selective degradation of a target via the proteasome or, less frequently, via chaperone-mediated autophagy [8, 97]. For example, a PROTAC was used to downregulate ER levels in breast cancer cells. Here, the PROTAC was composed of a methyl-ester of bestatin, which binds the cellular inhibitor of apoptosis protein-1 (IAP) and is linked to 4-hydroxy tamoxifen (4-OHT), which binds ER [98]. Under normal conditions, estrogen binding to ER leads to receptor translocation to the nucleus where it activates transcription [99]. However, the PROTAC induced cIAP1-mediated ubiquitylation and proteasomal degradation of ER in MCF7 cells, triggering necrotic cell death [98]. PROTAC technology also successfully decreased HER3 levels in breast cancer cells [100]. In the immediate future, it will be exciting to observe the effects of PROTACs in other cancer models and in animal models. Even with recent successes, the delivery and bioavailability of PROTACs remain the largest hurdles prior to clinical trials.

## Targeting the Hsp70 and Hsp90 molecular chaperones in breast cancer

The Hsp70 and Hsp90 heat shock proteins are among the most abundant and important house-keeping chaperones and are overexpressed in many cancers [61, 101–103]. These chaperones bind and release protein substrates via a complex ATP-regulated cycle and play central roles in multi-protein assemblies that mediate each event during protein folding, degradation, and activation (Fig. 1). Based on the fact that cancers amass misfolded proteins, and because they are over-expressed in some breast cancers, Hsp70 and Hsp90 have been targeted in breast cancer [104–107].

Hsp90 helps fold several oncogenic proteins, such as BRAF, HER2, AKT, and CREF [108–110], and elevated Hsp90 correlates with decreased survival in breast cancer patients [111]. Therefore, inhibition or knock down of Hsp90 is a viable target for cancer therapy, especially in HER2-positive breast cancers. Normally, HER2 is degraded after ligand-induced endocytosis [112, 113] and after binding select drugs [19].

A class of compounds developed to treat HER2-positive breast cancers includes derivatives of the benzoquinoid ansamycin antibiotic geldanamycin (GA; Table 2), which bind and inactivate Hsp90 and, in turn, induce HER2 degradation [20, 114, 115]. Hsp90 interacts with HER2 at the plasma membrane [109], and GA treatment leads to rapid HER2 downregulation by mediating ubiquitiylation and degradation [19, 115, 116]. It is unclear whether Hsp90 stabilizes HER2-containing heterodimers or if Hsp90-HER2 binding favors faster recycling to the plasma membrane in breast cancer cells. However, lapatinib, a tyrosine kinase inhibitor used to treat HER2-positive breast cancers, binds inactive HER2 and inhibits Hsp90–HER2 association to the same extent as GA and another analog, 17-AAG (Table 2). These data suggest that Hsp90 may be directly involved in dimer stabilization. On the contrary, lapatinib treatment does not induce HER2 degradation after ubiquitylation, which can occur via the action of another E3 ligase, CHIP; therefore, Hsp90 prevents CHIP recruitment [117, 118]. Recently, it was reported that kinase inhibitors, such as sorafenib (Nexavarand) and pazopanib (Votrient) (Table 2), were linked to the inhibition of Hsp90 activity in HER2-positive breast cancer cells [63]. Together, these results suggest that modulating HER tyrosine kinase activity, in conjunction with Hsp90, may become a valuable, new therapeutic strategy in breast cancer.

Even though promising pre-clinical data have emerged in HER2-positive breast cancers after treatment with Hsp90 inhibitors, in clinical trials no clear efficacy was detected. In fact, Hsp90 inhibition usually induces compensatory effects, such as increased expression of other heat shock proteins (Hsp70 and Hsp27) and activation of an anti-apoptotic response [61, 119–122]. In particular, upon Hsp90 inhibition, overexpression of Hsp27 was reported to modulate the oxidative stress response in cancer cells, and higher levels of Hsp70 were also detected, which helps block apoptosis induction and reduces the efficacy of Hsp90 inhibitors [120, 121]. Moreover, in patients Hsp90 inhibitor exposure is limited due to poor drug solubility and liver toxicity [25, 123].

One cellular Hsp90 partner is HSF1, a transcription factor involved in the cytoplasmic stress response (Fig. 1) [124, 125]. In the absence of stress, Hsp90 and other chaperones form a complex with HSF1 [125, 126]. However, Hsp90 inhibitors alter the composition of the HSF1-containing complex, which releases HSF1 and stimulates HSF1-activated transcription of heat shock proteins [125, 127]. As might be expected, then, HSF1 is associated with cancer progression [128]. To date, specific inhibitors of HSF1 have not been identified, but this represents an active area of research.

Cytosolic Hsp70 as well an ER lumenal Hsp70 homolog, BiP (or GRP78; see below), also play important roles in breast cancer, as well as in many other cancers. Hsp70 upregulation has been detected in different types of cancers, including endometrial cancer, osteosarcoma, and renal carcinoma [129, 130], and higher levels of Hsp70 are associated with metastasis and resistance to chemotherapy in glioblastoma and breast, endometrial, and cervical cancers [45, 131–133]. Hsp70 overexpression correlates with TNBC metastasis in murine models and human breast cancer patients [24], but knock down of cytosolic Hsc70 (a constitutive Hsp70) or Hsp70 (the inducible Hsp70) in breast cancer lines exhibits distinct responses, probably due to the relative expression levels of the two chaperones [61, 134]. However, dual knock down of the chaperones increased Hsp90 client ubiquitylation, that is, HER2, in ovarian cancer, suggesting a role for Hsp70 inhibitors in the treatment of HER2-positive breast cancer. Moreover, Hsp70 inhibition induces caspase-3 and PARP cleavage as well as sustained apoptosis in breast cancer cells [135]. The proposed mechanism by which Hsp70 inhibition triggers cancer cell death involves lysosomal membrane permeabilization [136–138]. Notably, Hsp70 binds bis-monoacylglycerol-phosphate, a membrane-bound anionic phospholipid that predominantly localizes to the inner lysosomal membrane, which maintains lysosomal membrane stability and cell survival. In rhabdomyosarcoma, on the other hand, cancer cell death mediated by Hsp70 inhibition was linked to UPR induction [139].

## The unfolded protein response in breast cancer: a protective pathway and a potential therapeutic target

As described above, the UPR is an adaptive mechanism that restores endoplasmic reticulum homeostasis [28, 30, 65]. There are three UPR branches that are coordinately activated in mammalian cells: the inositol-requiring enzyme 1 (IRE1), PKR-like endoplasmic reticulum kinase (PERK), and activating transcription factor 6 (ATF6) branches [28, 140] (Fig. 1). Under normal conditions, PERK, IRE1, and ATF6 are maintained in an inactive form in the endoplasmic reticulum membrane by binding to BiP (also known as glucose-regulated protein 78 (GRP78)), which also recognizes unfolded secretory proteins with exposed hydrophobic patches and helps target misfolded proteins for proteasomal degradation via the ERAD pathway [141–143]. As described later, BiP is also an important therapeutic target in cancer. During endoplasmic reticulum stress, BiP is released from PERK, IRE1, and ATF6 and binds misfolded proteins that begin to accumulate [144]. As a result, PERK phosphorylates eIF2α, inhibiting protein translation and leading to the expression of activating transcription factor 4 (ATF4) [145]. Higher levels of ATF4 in turn increase the levels of CHOP, a pro-apoptotic transcription factor (also known as DDIT3) and growth arrest and DNA damage-inducible protein 34 (GADD34). Both ATF4 and CHOP also upregulate the transcription of autophagy-related genes (ATGs) [146] and, after a prolonged response, induce cell death [147, 148]. In contrast, ATF6 is transported to the Golgi during endoplasmic reticulum stress and is cleaved to liberate a soluble, active transcription factor that induces the production of chaperones and redox related proteins, including BiP, GRP94, PDIA4, and PDIA6 [149–151]. Finally, the release of BiP from IRE1 favors receptor oligomerization and autophosphorylation. In addition to its kinase activity, IRE1 is an endonuclease that catalyzes splicing of the X-box binding protein 1 (XBP1) message. Once the message is spliced and translated, the resulting Xbp1 transcription factor activates the synthesis of chaperones, ERAD components, and inflammatory responsive genes [151–153].

UPR induction can be either protective or deleterious for cell survival, depending on the cancer. In fact, the UPR can even act as a pro-tumorigenic signal, increasing tumor cell protein folding capacity and drug resistance [154]. However, prolonged endoplasmic reticulum stress activates cell death pathways, such as mitochondrial-associated apoptosis [155, 156] and CHOP-dependent cell death (see above) [144, 157, 158]. Examples of these disparate functions include the fact that high levels of Xbp1 are present in some breast cancer tissues, and a greater amount of the spliced XBP1-message correlates with poor prognosis in TNBCs [159, 160]. It is also known that HIF1α, the hypoxia-inducing factor 1α, is hyperactive in TNBCs [161, 162]. A genome-wide map of the Xbp1 regulatory network in TNBCs suggested that Xbp1 tumorigenicity is assisted by the formation of a complex with HIF1α [162]. Moreover, an analysis of independent cohorts of patients with TNBC revealed that a spliced XBP1 signature correlated with HIF1α and hypoxia-associated signatures, underlying the importance of the IRE1/Xbp1 signaling pathway in TNBC. In addition, depletion of Xbp1 in TNBC cell lines inhibits tumor growth and relapse [162].

Another example in which the UPR can be either a pro-survival or a cell death signal is in the epithelial to mesenchymal transition (EMT). The EMT favors tumorigenesis and drug resistance in mammary gland tumors [163, 164]. In human tumor tissues, EMT gene expression correlates with PERK–eIF2α responsive genes, but not with other branches of the UPR [163].

Endocrine therapies (such as tamoxifen and fulvestrant; Table 1) remain a mainstay for the treatment of ER-positive breast cancer patients. Although there is an initial positive response of a ~ 70% reduction in tumor volume (in treated patients), acquired resistance is ultimately evident in about half of all patients [28]. Interestingly, a rapid “anticipatory” UPR that is independent of endoplasmic reticulum stress is induced upon ligand binding to ER. By contrast, long-term treatments with tamoxifen and fulvestrant cause endoplasmic reticulum stress and induce the UPR, promoting cancer survival and drug resistance in ER-positive breast cancers [165–169].

Anti-estrogen-resistant breast cancer cell lines express elevated levels of BiP and Xbp1 [170–172], suggesting that UPR induction contributes to therapy resistance. One compound, HA15, targets BiP, which is one of the most highly induced targets of the UPR and plays a central role in UPR signaling (see above; Fig. 1). HA15 is effective in a breast cancer cell line and in other tumors, such as melanomas, and kills these cells after inducting an endoplasmic reticulum stress response [173]. These and other studies indicate that modulation of BiP activity and the UPR should be further coopted as therapeutic targets.

As mentioned above, the initiation factor eIF2α is phosphorylated by PERK, which attenuates the synthesis of new proteins and reduces the concentration of unfolded proteins in the endoplasmic reticulum [145, 174]. However, eIF2α can also be phosphorylated by other kinases, such as GCN2 (general control non-depressible 2), PKR (interferon-inducible dsRNA-activated protein kinase), and HRI (heme-regulated inhibitory) [175–178]. During induction of the integrate stress response (ISR)—which is a specialized form of the UPR—GCN2-dependent eIF2α phosphorylation occurs after amino acid starvation, which also induces ATF4 [179]. In this case, ATF4 enhances the expression of autophagy-requiring genes (for example ATG5, 7, and 10) that facilitate cellular recovery after starvation. Consistent with these data, GCN2 silencing decreases cancer cell survival after amino acid starvation and attenuates tumor growth in murine models [180]. Elevated levels of ATF4 also correlate with resistance to chemotherapeutic agents, including DNA damaging agents (ionizing radiation, aphidicolin, hydroxyurea, cytarabine, etoposide, doxorubicin, and mafosfamide), celecoxib (a nonsteroidal anti-inflammatory drug), and bortezomib, in different cancers [181–186]. These data suggest that differential sensitivities to proteasome inhibitors, like bortezomib, in breast cancer and multiple myeloma could be due to selective activation/upregulation of UPR components. Together, the UPR and ISR enable cancer cells to integrate multiple stress stimuli into a common control mechanism, centering on eIF2α phosphorylation and, depending on the duration and magnitude of the stress, acting as either a pro- or anti-tumorigenic signal [30]. Consequently, studies on UPR modulation are ongoing in breast cancer cells as well as in other cancers. For example, a small molecule, BHPI, was used to demonstrate that hyperactivation of the PERK branch of the UPR blocks proliferation of ER-positive breast, ovarian, and endometrial cancer cells due to persistent inhibition of protein synthesis [168, 187, 188].

## BiP upregulation is a marker for drug resistance in breast cancer

The endoplasmic reticulum folding capacity is in part limited by the chaperone reservoir. The expression of chaperones like BiP, GRP94, and calreticulin are tightly regulated [45, 47, 189, 190]. BiP was first discovered as a glucose responsive protein (hence the alternative name, GRP78) as well as via its association with immunoglobulin heavy chain [191–193]. In addition, as described above, BiP plays a crucial role during UPR induction [144, 194, 195]. BiP protein levels are normally maintained at moderate levels in adult tissues such as brain, lung, and heart, but this chaperone is strongly induced in breast, melanoma, colon, and adenocarcinoma cancer cells [196–200]. In many cases, BiP expression is associated with poor prognosis and chemotherapy resistance [201–205]. A retrospective study also examined if BiP can be used as a marker of chemotherapy resistance in tumors. As hypothesized, one-third of breast cancer patients have high levels of BiP before treatment, and the risk of recurrence was greater in chemotherapy-treated patients with moderate to high BiP levels [202].

Even though BiP is an endoplasmic reticulum resident, where it regulates the UPR and catalyzes protein folding and ERAD, recent studies demonstrate that this chaperone can also reside at the cell surface, as well as in the cytosol, mitochondria, and nucleus; these data and other results suggest that BiP performs a novel function to regulate cell proliferation, invasion, and apoptosis [206–208]. More generally, a cell surface proteomic analysis of tumor cells confirmed that several cytosolic heat shock proteins and endoplasmic reticulum chaperones, like BiP, reside on the extracellular surface of the plasma membrane, suggesting that chaperone relocation may be an adaptive response to stress induced by perturbations in proteostasis [38, 208]. Moreover, cell surface-resident BiP is more abundant in pancreatic and breast cancers [208].

The extracellular pool of BiP is associated with specific membrane glycoproteins and its amount is enriched after ER stress [209, 210]. As anticipated for a bona fide chaperone-based interaction, the substrate-binding site in BiP is required to interact with these partners [208]. However, the mechanism by which BiP translocates to the extracellular face of the plasma membrane is unclear. Nevertheless, UPR induction favors BiP secretion, possibly due to its overexpression, which in turn may overwhelm the machinery that retains proteins in the secretory pathway [211].

Several studies have demonstrated that BiP also binds BIK, a pro-apoptotic member of the Bcl2 family, impairing apoptotic cell death in ER-positive tumors [212]. The pro-apoptotic Bcl2 family members facilitate the release of cytochrome c from the mitochondrial membrane to the cytosol, which induces the apoptotic cascade [213–215]. In contrast to other pro-apoptotic Bcl2 members that reside in the mitochondria, BIK is an endoplasmic reticulum membrane protein and does not interact directly with pro-apoptotic family members, such as BAX and BAK [214, 216]. BIK plays a critical role in promoting estrogen starvation or anti-estrogen-mediated cell death in human breast cancer cells [217], and BIK knock down impairs estrogen starvation-induced cell death in MCF7 cells [212]. Recent studies suggest that BiP upregulation suppresses BIK activity by inhibiting the apoptotic response after anti-estrogen therapies in ER-positive breast cancer [212, 218]. For this reason, the development of specific BiP inhibitors represents an important goal. In fact, a natural product of the naphthoquinone family, plumbagin, was identified that initiates cell death in ER-positive breast cancer cells by upregulating BIK levels [219, 220]. Plumbagin-mediated BiP inhibition also sensitized breast cancer cells to tamoxifen-mediated cell death. In addition, BiP knock down impaired the plumbagin-mediated increase in Bik, suggesting an inhibitory role of this compound on BiP-mediated downregulation of BIK in breast cancers [220]. Overall, BiP is emerging as a novel target to predict cancer outcomes and therapeutic options [209, 221–223].

## The role of autophagy in breast cancer

As outlined in the preceding sections, the autophagy pathway degrades misfolded and aggregating proteins in the cytoplasm, thereby helping to maintain cellular homeostasis after cellular stress. Autophagy also provides a mechanism to replenish cellular energy stores during starvation [38–44, 224]. The autophagy pathway is associated with a variety of processes such as tumor suppression, aging, development, and microorganism elimination [41, 45]. The dependence of cancer cells on autophagy is dictated in part by the nature and the length of the stress [225]. For example, UPR induction together with autophagy upregulation are linked to endocrine tumor therapy resistance [158, 170, 226–228]. More specifically, autophagy inhibition increases the anti-estrogen response in tumors [166, 167, 229–233], and UPR induction promotes autophagy [146, 234, 235]. In addition, UPR induction, and in particular PERK activation, increases eIF2α phosphorylation and the translation of select mRNAs, like ATF4 [236–238]. ATF4 then triggers the expression of genes involved in the ISR as well as those required for autophagy [178, 184, 239–241] (also see above).

Several studies have demonstrated that lapatinib- or trastuzumab-resistant HER2-positive cancer cell lines can be obtained by upregulating the autophagy pathway [242, 243]. Interestingly, ATG12, a protein required for autophagosome elongation, was induced in HER2-positive resistant cells and its downregulation compromised acquired resistance [243, 244]. Up-regulation of ATG5, which is covalently attached to ATG12 during autophagy, also facilitated lapatinib resistance in HER2-positive cells [245]. Therefore, inhibition of these autophagic components might sensitize HER2-positive cancer cells to established treatments.

Autophagy also plays an important role during metastases in neoplastic breast cancer [246, 247]. Chloroquine and chloroquine derivatives (which inhibit autophagolysosome formation), as well as Bcl2 inhibitors (which inhibit early steps in the autophagy pathway), are currently in clinical trials to treat metastatic/invasive breast cancer, ductal carcinomas, and HER2-positive cancers [248–250].

Recently, a correlation between autophagy and the expression of Runx2, a Runt-related transcription factor involved in cell survival during metabolic stress and breast cancer progression, was uncovered [247]. Runx2 promotes the metastatic spread of mammary tumors to the bone, which is a recurrent location for TNBC and luminal breast cancer metastasis [251]. Indeed, aberrant Runx2 expression in metastatic breast cancers altered the activity of PI3K, mTORC1, and AMPK, which function as upstream modulators of autophagy [251–255]. Misregulation of PI3K/Akt/mTOR pathways is often associated with endocrine resistance in ER-positive breast cancers [3]. As might be anticipated, then, Runx2 knock down in a breast cancer cell line reduced metastatic dissemination, suggesting a link between Runx2 and autophagy [247].

A microtubule associated deacetylase, known as HDAC6, has also been suggested to link Runx2 and autophagosome formation/lysosomal fusion [247]. HDAC6 binds polyubiquitylated, misfolded proteins and couples them to the dynein motor complex, facilitating the trafficking of aggregated proteins to autophagosomes [235, 256]. In the absence of stress, HDAC6 is associated with p97, Hsp90, and HSF1 (Fig. 1) [126]. In turn, p97 function is associated with HDAC6-dependent fusion of aggresomes—which are microtubule-associated clusters of ubiquitylated and aggregated proteins—with autophagosomes [257, 258]. Based on its protein “segragase” activity, p97 dissociates HDAC6 from polyubiquitylated proteins and regulates HDAC6 shuttling, governing both proteasome- and autophagy-dependent clearance of misfolded proteins [259–261]. In solid tumors, such as breast cancer, the use of a proteasome inhibitor has minimal effect [262, 263]. However, in other cancers, such as multiple myeloma, inhibition of both proteasome activity and HDAC6 using tubacin synergistically promote cell death through the accumulation of toxic polyubiquitylated protein aggregates [256, 264, 265]. These data suggest again that inhibition of both proteasome and autophagy-mediated degradation could be beneficial in some cancers.

Consistent with this view, treatment with the proteasome inhibitor bortezomib in a breast cancer cell line increased the levels of LC3B, a marker of autophagy, at both the protein and mRNA levels [235]. However, ATF4 knock-down limited LC3B induction after bortezomib treatment, confirming the importance of ATF4 as a mediator of the compensatory response in ER-positive cancer cells. In turn, HDAC6 is also linked to bortezomib resistance in MCF7 cells: Knock down of HDAC6 led to a synergistic effect on MCF7 cell death after bortezomib treatment [164, 266]. These data indicate that HDAC6 may be an important therapeutic target when proteasome activity is impaired. As new and improved inhibitors of the proteasome come on-line, synergistic effects of these compounds with specific HDAC6 inhibitors—and other autophagy inhibitors—should be assessed for their efficacy in breast cancer cells [267, 268].

## Open questions and new therapeutic approaches in breast cancer

Based on emerging discoveries from basic research programs and from clinical trials, it is clear that protein quality control and stress response pathways are crucial for cancer cell survival. Therefore, the targeting of these pathways will not only improve patient treatment but also help answer fundamental questions related to cancer biology. To this end, novel compounds that modulate proteostasis pathways and cellular stress responses, such as the UPR, are currently being evaluated in cell and rodent models [14, 27, 29, 30, 80, 269, 270].

Even with these recent developments, numerous questions remain. For example, the sensitivity of different cancers (even within distinct cancer sub-types) to proteasome inhibitors is quite heterogeneous, and in some cases the lack of a correlation between preclinical models and patient outcomes remains mysterious [269]. Thus, it is vital that we define the factors and pathways that give rise to these phenomena. It is also important to identify novel biomarkers that predict drug efficacy, and in particular the efficacy of proteasome inhibitors [29]. One such biomarker might be the extent of autophagy induction, as discussed above, and studies with solid tumors suggest that autophagy is a source of bortezomib resistance [271, 272].

A second question relates to the dual role of UPR induction as either a pro-survival or a pro-death signal in cancer [146, 184, 241]. The recently discovered anticipatory UPR, which can be induced by ER signaling in breast cancer cells, is an intriguing candidate pathway to treat ER-positive breast cancer patients. At the same time, however, patients treated with endocrine therapies might not benefit from this potential treatment.

Nevertheless, induction of the PERK branch of the UPR is associated with increased autophagy [239–241]. These data suggest that a combination of proteasome and PERK inhibitors, which place an increased burden on the proteasome, may sensitize tumors to canonical treatments. This would also allow for lower drug dosages, which may help avoid potential side effects and decrease the likelihood of drug-resistant point mutations in HER and ER [30]. However, it is unknown if blocking UPR-induced autophagy or inhibiting upstream UPR effectors will prove more effective. Regardless, combinatorial treatments remain the best option to avoid cancer cell resistance and cancer relapse [273], especially since most therapeutic treatments for HER2-positive breast cancers (radiotherapy, chemotherapy and HER2 inhibitors) activate autophagy [249, 250].

A third question is why markers to predict chemotherapy or radiotherapy effectiveness have not been fully verified [220]. However, as outlined in this review, cell surface BiP is a prime candidate to predict chemotherapy effectiveness. On the contrary, increased expression of BiP in neuroblastoma patients correlates with longer survival [274]. These contradictory findings in human clinical samples highlight the necessity for further studies on the role of BiP in different tumorigenic contexts. It is also important to consider whether induction of intra- versus extracellular BiP hint at different outcomes. Another problem in identifying biomarkers is that metastasis formation can occur at early stages or during late stage tumor development [275, 276]. Metastases arising from late stage primary tumors are more heterogeneous, which favors chemotherapeutic resistance.

Finally, it is worth noting that drug discovery efforts and clinical trials to modulate the activity of distinct components of the “proteostatis network”, including components of the proteasome-ubiquitin machinery, Hsp90, p97, and the autophagy pathway are in progress [86, 267, 268, 270, 277–286]. When combined with standard and emerging therapies for each breast cancer cell sub-class, we envision that synergistic effects to improve clinical outcomes for patients will then become evident [8, 224, 287, 288].
